# Profibrotic role of WNT10A via TGF-β signaling in idiopathic pulmonary fibrosis

**DOI:** 10.1186/s12931-016-0357-0

**Published:** 2016-04-12

**Authors:** Keishi Oda, Kazuhiro Yatera, Hiroto Izumi, Hiroshi Ishimoto, Sohsuke Yamada, Hiroyuki Nakao, Tetsuya Hanaka, Takaaki Ogoshi, Shingo Noguchi, Hiroshi Mukae

**Affiliations:** Department of Respiratory Medicine, University of Occupational and Environmental Health, Japan, 1-1, Iseigaoka, Yahatanishiku, Kitakyushu City, Fukuoka 807-8555 Japan; Department of Occupational Pneumology, University of Occupational and Environmental Health, Japan, 1-1 Iseigaoka, Yahatanishiku, Kitakyushu City, Fukuoka 807-8555 Japan; Department of Pathology and Cell Biology, University of Occupational and Environmental Health, Japan, 1-1, Iseigaoka, Yahatanishiku, Kitakyushu City, Fukuoka 807-8555 Japan; Department of Pathology, Field of Oncology, Graduate School of Medical and Dental Sciences, Kagoshima University, 8-35-1 Sakuragaoka, Kagoshima, 890-8544 Japan; Miyazaki Prefectural Nursing University, 3-5-1 Manabino, Miyazaki City, Miyazaki 880-0929 Japan; Second Department of Internal Medicine, Nagasaki University School of Medicine, 1-7-1 Sakamoto, Nagasaki, 852-8501 Japan

**Keywords:** Acute exacerbation, Idiopathic pulmonary fibrosis, Autopsy, Diffuse alveolar damage, WNT/β-catenin pathway, Fibroblast

## Abstract

**Background:**

WNT/β-catenin signaling plays an important role in the pathogenesis of idiopathic pulmonary fibrosis (IPF); however, the role of WNT10A via transforming growth factor (TGF)-β signaling remains unclear.

**Methods:**

We evaluated the expression of WNT10A and TGF-β in bleomycin (BLM)-treated mice and the interactions between TGF-β or BLM and WNT10A in vitro. Additionally, we investigated IPF patients who underwent video-assisted thoracoscopic surgery to determine whether the WNT10A expression is related to the survival.

**Results:**

Increased WNT10A and TGF-β expressions were noted in the BLM-treated mice. Real-time PCR and luciferase reporter assays demonstrated the levels of WNT10A and collagen in the fibroblasts cells to increase after TGF-β administration. Conversely, WNT10A siRNA treatment inhibited the synthesis of collagen in the transfected fibroblasts cells. A Kaplan-Meier survival analysis demonstrated a tendency toward a poor survival among the IPF patients with a WNT10A-positive expression compared to those with a negative expression (Hazard ratio 5.351, 95 % CI 1.703-16.82; *p* = 0.0041). An overexpression of WNT10A was found to be significantly predictive of an acute exacerbation of IPF (AE-IPF) (Odds ratio 13.69, 95 % CI 1.728-108.5; *p* = 0.013).

**Conclusions:**

WNT10A plays an important role in the pathogenesis of IPF via TGF-β activation and it may also be a sensitive predictor for the onset of an AE-IPF.

## Background

Idiopathic pulmonary fibrosis (IPF) is a progressive and usually fatal lung disease of unknown etiology for which no effective treatment currently exists [1, 2]. This progressive disease has a high mortality rate, and current models for predicting mortality have limited value in identifying which patients will progress. The concept of acute exacerbation of idiopathic pulmonary fibrosis (AE-IPF) has been previously recognized [3], and the findings for several cohorts suggest that this condition is a major cause of death in patients with IPF [4, 5]. However, the molecular mechanisms and biomarkers for predicting AE-IPF are currently only partially understood.

The histopathological features of patients with IPF include the presence of fibroblastic foci of fibroblasts and myofibroblasts consisting of aggregates of activated fibroblasts that produce excessive levels of extracellular matrix components within the alveolar space at sites of epithelial cell loss. Lung tissues derived from patients with IPF show an increased activation response to fibrogenic cytokines, such as transforming growth factor (TGF)-β1 [6]. The epithelial-mesenchymal transition (EMT) of alveolar epithelial cells is widely observed in patients with IPF. Furthermore, TGF-β is a major inducer of the EMT and a key mediator of fibrosis in the lungs.

WNTs constitute a family of secreted glycoproteins that consist of at least 19 ligands in mammals and mediate autocrine and paracrine effects by binding to frizzled receptors and LDL-related protein 5/6 co-receptors. Different WNT ligands signal through the canonical β-catenin-dependent pathway or non-canonical β-catenin-independent pathway. Although WNT signaling pathways have been shown to function in the setting of pulmonary fibrosis [7–10], the relationship with WNT10A is not well defined. We previously reported that WNT10A is expressed in dermal and kidney fibroblasts [11, 12]. Moreover, WNT10A overexpression increases the expression of fibronectin at sites of these lesions. Furthermore, recent studies have shown that the interactions between TGF-β and WNT are crucial for many biological processes [13]. Therefore, we hypothesized that the WNT10A expression, which is required for TGF-β, plays an important role in tissue repair and fibrotic processes associated with IPF.

In this study, we evaluated the expression of WNT10A in the setting of bleomycin (BLM)-induced lung fibrosis in mice and assessed the interactions between TGF-β, BLM and WNT10A at sites of fibroblast cells in vitro. In addition, we investigated the prognosis and clinical implications of WNT10A overexpression.

## Methods

### Animals

Experiments were performed in seven- or eight-week-old male C57BL/6 J mice (wild-type mice) (Kyudo, Co., Ltd., Tosu, Japan) weighing 21–25 g. The mice were maintained on a regular diet (CE-2, CLEA Japan, Inc., Tokyo, Japan). This study was reviewed and approved by the Ethics Committee of Animal Care and Experimentation, University of Occupational and Environmental Health, Japan, and were performed in accordance with the National Institutes of Health guidelines. The mice were divided into two groups (a bleomycin (BLM) group and control group, *n* = 10/group). The mice received intraperitoneal pentobarbital sodium and were intratracheally administered 2.0 mg/kg of BLM (Nippon, Kayaku, Tokyo, Japan) dissolved in 50 μl sterile saline or 50 μl sterile saline alone as a control. The body weights were measured daily beginning on the day when BLM or sterile saline was administered.

### Bronchoalveolar lavage (BAL) and sample collection

On day 14, the mice were anesthetized by an injection of pentobarbital sodium. Immediately thereafter, a midline neck incision was made, and the trachea was cannulated. The left lung was lavaged using 2.4 mL cooled sterile saline, 0.8 mL each time, for a total of three times. The brochoalveolar lavage fluid (BALF) obtained from these three washes were collected together. After counting the cell numbers in the BALF, the cells were cytospun and stained with Diff-Quick for cell classification according to the manufacturer’s protocol.

The right lung was harvested with RNAlater RNA Stabilization Reagent (QIAGEN, Germantown, MD, USA) and frozen at -70 °C for Real-Time quantitative polymerase chain reaction (qPCR). The remaining left lungs were fixed in 15 % formalin neutral buffer solution (Wako, Osaka, Japan) and embedded in paraffin before a histological analysis.

### Real-Time qPCR

One μg of total RNA was reverse-transcribed according to the manufacturer’s protocol. Quantification of the expression levels of TGF-β1, monocyte chemotactic protein (MCP)-1, collagen, type I, alpha 1 (COL1A1), WNT3A, WNT5A, WNT7B, WNT10A, WNT10B and glyceraldehyde-3-phosphate dehydrogenase (GAPDH) mRNA was performed by real-time qPCR on an ABI prism 7000 sequence detection system (Applied Biosystems, Foster City, CA). The following TaqMan Gene Expression primers were used: TGF-β1 (Mm01178820_m1), MCP-1 (Mm00446214_m1), COL1A1 (Mm00801666_g1 and Hs00164004_m1), WNT3A (Hs00263977_m1), WNT5A (Hs00998537_m1), WNT7B (Hs00536497_m1), WNT10A (Mm00437325_m1 and Hs00228741_m1), WNT10B (Hs00559664_m1) and GAPDH (Mm99999915_g1 and Hs02758991_g1).

### Histopathological evaluation of tissue sections

Tissue sections (4-μm thick) were cut and stained with hematoxylin and eosin (H&E), an anti-α-SMA antibody and Masson’s trichrome using standard procedures. We evaluated the effects of BLM-induced pulmonary fibrosis by means of the Ashcroft score [14]. In addition, to investigate the expression of WNT10A from the morphological perspective in BLM-induced pulmonary fibrosis, an immunohistochemical analysis was performed.

### Cell culture and treatment

The normal human lung fibroblast cell line, IMR-90 (Japanese Collection of Research Bioresources, Osaka, Japan), and the IPF lung fibroblast cell line, LL97A (American Type Culture Collection, Manassas, VA, USA), were cultured in Eagle’s Minimum Essential Medium (MEM) with Earle’s salt (GIBCO) supplemented with 10 % fetal bovine serum, 100 U/ml of penicillin, 100 μg/ml of streptomycin and 0.1 mM nonessential amino acids. The African green monkey fibroblast line, COS1 (American Type Culture Collection, Manassas, VA, USA), was also cultured as described previously [12]. IMR-90 and LL97A cells were plated in six-well plates and stimulated for 24 h with recombinant TGF-β1 (5 ng/ml) (R&D Systems, Inc., Minneapolis, USA). We performed a Real-Time qPCR analysis to examine the time dependency of the expression profiles of the WNT ligands and COL1A1 by stimulation with TGF-β1, and performed the Sircol collagen assay to quantify the total collagens. In addition, whole-cell lysates were prepared and subjected to Western blot analyses.

### Antibodies

For the Western blotting analyses and immunohistochemical studies, the following antibodies were used: anti-WNT10A (SAB3500393) and anti-β-actin (A5316) were purchased from Sigma-Aldrich (MO, USA), anti-α-SMA (M851) was purchased from Dako (Tokyo, Japan), and anti-β-catenin (9562) was purchased from Cell Signaling Technology (MA, USA).

### Plasmid construction and transfectants

To prepare the human WNT10A-luciferase reporter plasmid, the pGL3 human WNT10A promoter-luciferase plasmid [12], pEBMulti-vector (Wako, Osaka, Japan) and pMACS Kk.II (Miltenyi Biotec, Bergisch Gladbach, Germany) were modified. This plasmid was transfected into COS1 cells. In the COS1 cells, EBNA1, H-2Kk, EmGFP and hygromycin were all expressed. The EBNA1 and OriP sequences could replicate this plasmid in COS1 cells. After 72 h, transfected cells were separated using an anti-H-2Kk antibody conjugated to magnetic beads based on the manufacturer’s instruction manual, and were cultured in medium containing 400 ng/ml hygromycin for two weeks. The transfection efficiency was estimated based on the EmGFP expression in living cells.

### Reporter assay

The reporter assay was described previously [12]. In brief, 4x10^4^ human WNT10A-luc transfectants in 12 well plates were cultured for 12 h, then were treated with BLM (0 to 50 uM) or 5 ng/ml TGF-β1 for 24 h. For the recovery assay, 4x10^4^ human WNT10A-luc transfectants were treated with 50 uM BLM for the indicated time, and were cultured with new medium without BLM for 48 h. The results shown were normalized to the protein concentrations measured using the Bradford method and are representative of at least three independent experiments.

### Western blot analysis

Cells were lysed using RIPA buffer containing 50 mM Tris, 150 mM NaCl, 1.0 % NP-40, 0.5 % sodium deoxycholate, 1.0 mM EDTA, 0.1 % SDS and 0.01 % sodium azide at a pH of 7.4 (Rockland, Gilbertsville, PA, USA), plus a mix of protease inhibitors (Nakalai tesque, Kyoto, Japan). The protein concentration was analyzed using a protein assay kit, with bovine serum albumin used as the standard (Thermo Fisher Scientific, Rockford, IL, USA). A 30 μg aliquot of total protein was typically used for the Western blot analyses. The 30 μg of protein from whole-cell lysates were separated by sodium dodecyl sulfate-polyacrylamide gel electrophoresis and transferred to polyvinylidene difluoride membranes (Bio-Rad, USA). The blotted membranes were treated with 5 % (*w/v*) skimmed milk in 10 mM Tris, 150 mM NaCl and 0.2 % (*v/v*) Tween 20, and were incubated for 1 h at room temperature with the primary antibody. An anti-β-actin antibody was used as an internal control at a 1:1,000 dilution. The membranes were then incubated for 45 min at room temperature with a peroxidase-conjugated secondary antibody, and were visualized using an ECL kit (GE Healthcare Bio-Science, Buckinghamshire, UK). The chemiluminescence was detected with the Light-Capture AE-6972 (ATTO, Tokyo, Japan). Western blots were scanned densitometrically using the Image J software program.

### Small interfering RNA transfection

Specific knockdown was achieved using small interfering RNA against WNT10A (sc-76927) or a control siRNA (sc-37007) (all from Santa Cruz Biotechnology, Inc.). IMR-90 and LL 97A cells were transfected with WNT10A siRNA for 72 h according to the manufacturer’s protocol. Cells were harvested for 24 and 48 h (for mRNA and protein determination, respectively.)

### Sircol collagen assay

IMR-90 or LL97A cells were plated at a density of 10,000 cells/well in six-well plates, synchronized for 24 h in serum-free medium, and treated for 24 h as indicated. The total collagen content was determined using a Sircol Collagen Assay Kit (Biocolor Ltd., Carrickfergus, Northern Ireland, U.K.) according to the manufacturer’s instructions. Briefly, equal volumes of conditioned medium were incubated with Isolation and Concentration Reagent at 4 °C overnight. Samples were then centrifuged at 12,000 rpm for 10 min using a microcentrifuge, and Sircol Dye Reagent was then added to the pellet and incubated with shaking for 30 min. Samples were centrifuged as above to allow for packing of the collagen-dye complex. The supernatant was discarded, and cold Acid-Salt Wash Reagent was added without disrupting the pellet. Samples were centrifuged as above, the supernatant was discarded and Alkali Reagent was added to the pellet. Samples were vortexed and then the absorbance was measured at 595 nm using a spectrophotometer. The total collagen concentration was estimated using a collagen standard.

### Immunofluorescence staining

IMR-90 cells were plated onto chamber slides for 72 h and fixed with 15 % formalin for 10 min after washing with PBS. The cells were incubated with 0.2 % (*v/v*) Triton X-100 with PBS for 10 min at room temperature. The cells were then treated with 4 % (*w/v*) skimmed milk (DS Pharma Biomedical Co., Ltd., Osaka, Japan) in PBS and incubated for 30 min at room temperature, washed with PBS and incubated for 1 h at room temperature with an anti-WNT10A antibody and subsequently with an Alexa Fluor 546-conjugated secondary antibody (red) (Invitrogen, Carlsbad, CA, USA) for an additional 30 min. For actin filament staining, phalloidin Alexa Fluor 488 (green) (Lonza, Walkersville, USA) was used, and for nuclear staining, Hoechst 33258 (blue) (Dojindo, Kumamoto, Japan), was used.

### Preparation of human lung samples

Thirty patients with IPF diagnosed according to the criteria of the 2011 IPF guidelines [15] who consecutively underwent video-assisted thoracoscopic surgery (VATS) lung biopsies at our university hospital from January 1, 2006 to December 31, 2012 were retrospectively analyzed. All cases were originally diagnosed histologically as diagnostic of usual interstitial pneumonia (UIP) by two pulmonary pathologists. This study was approved by the University of Occupational and Environmental Health, Japan Institutional Review Board (H26-004). We retrospectively studied the various data of the patients with IPF, including their history of AE-IPF. AE-IPF was defined according to a previous report [3].

### Immunohistochemistry of human lung tissue samples

Immunohistochemical staining was performed according to the antibody-linked dextran polymer method for antibody-bridge labeling, with hematoxylin counterstaining (EnVision; DAKO Cytomation Co., Glostrup, Denmark). Deparaffinized and rehydrated 4-μm sections were incubated in 10 % H_2_O_2_ for five minutes to block the endogenous peroxidase activity. The sections were subsequently rinsed and incubated with rabbit polyclonal anti-WNT10A (diluted 1:100) antibodies for 30 min. The second antibody-peroxidase-linked polymers were then applied, and the sections were incubated with a solution consisting of 20 mg of 3.3′-diaminobenzidine tetrahydrochloride, 65 mg of sodium azide and 20 ml of 30 % H_2_O_2_ in 100 ml of Tris-HCL (50 mM, pH7.6). After counterstaining with Meyer’s hematoxylin, the sections were observed under a light microscope. The sections were first scanned at low-power for all fields (original magnification: × 40) with UIP and non-UIP, respectively, to account for the heterogeneity of the distribution. The sections stained with WNT10A were then counted at high-power (original: × 400) magnification. The number of fibroblastic stromal cells within fibroblastic foci showing positive cytoplasmic staining and the pattern of staining were recorded. Necrotic tissues and lymphoid cells were not included in the recording. Immunoreactivity for WNT10A in each case was assessed semi-quantitatively by evaluating the proportion of positive fibroblastic cells relative to the total number of fibroblastic stromal cells in 5 % increments (0 %, 5 %, 10 %…). We selected and validated immunohistochemical cut-off scores for WNT10A positivity (10 %) based on the performance of a receiver operating characteristic (ROC) curve analysis. According to the ROC curve, the patients were divided into two groups, as follows: positive for more than 10 % positivity and negative for less than 9 % positivity.

All histological and immunohistochemical slides were evaluated by two independent observers (certified surgical pathologists at the Department of Pathology) using a blind protocol design (the observers were blinded to the clinicopathological data). The degree of agreement between observers was excellent (>0.9) for all antibodies investigated, as measured by the interclass correlation coefficient. In the few instances of disagreement, a consensus score was determined by a third board-certified pathologist in our department.

### Statistical analysis

The data are presented as the mean ± SEM. Differences between two groups were analyzed for statistical significance using Student’s *t*-test. The probability of overall survival was estimated using the Kaplan-Meier method and compared using the log-rank test. Correlation coefficients were calculated using Spearman’s rank correlation coefficient. The associations between acute exacerbation within one year on VATS and the explanatory variables were examined using a multiple logistic regression analysis adjusted for the effects of confounding variables. In the logistic regression, we selected the set of variables to be included in the model according to the backward elimination method. All statistical analyses were performed using the Statistical Package for Social Sciences (SPSS, version 19). Statistical values of *p* < 0.05 were considered to be significant.

## Results

### Analysis of pulmonary inflammation and fibrosis in BLM-treated mice

**Fig. 1:**
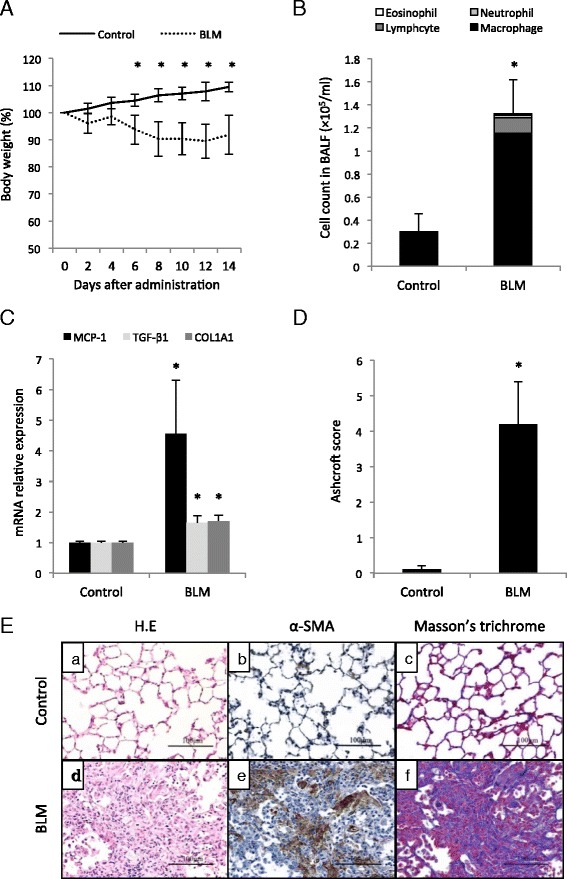
Inflammation in BLM-treated mice **a** The body weight changes of BLM-treated mice and control mice over time (*n* = 10 each). *p < 0.05. **b** The results of an analysis of the total cell numbers and cell types in the bronchoalveolar lavage fluid (BALF) (*n* = 10 each). *p < 0.05. **c** The expression levels of MCP-1, TGF-β and COL1A1 mRNA were increased in the lungs from the BLM-treated mice compared to the control mice (*n* = 6 each). *p < 0.05. **d** The Ashcroft score in the BLM-treated mice was significantly increased compared to that of the control mice (*n* = 4 each). *p < 0.05. **e** Representative photomicrographs of lung tissue sections stained with H&E, Masson’s trichrome or with an anti-α-SMA antibody. All results are expressed as the means ± SEM

We first evaluated the pulmonary inflammation and fibrosis in the BLM-treated mice. The BLM-treated mice lost more weight than the saline-treated mice (control mice) (Fig. 1a). After 14 days, the number of inflammatory cells in the BAL fluid (BALF) was increased in the BLM-treated mice (Fig. 1b). A real-time qPCR analysis showed more abundant MCP-1, TGF-β1 and COL1A1 mRNA in the homogenates of lungs from BLM-treated mice compared to those of the control mice (Fig. 1c). Serial lung sections from mice obtained after BLM treatment for 14 days were stained with H&E, and α-SMA antibody and Masson’s trichrome stain. The Ashcroft score in the BLM-treated mice was significantly increased compared to that in the control mice (Fig. 1d). Representative histological sections from the lungs of mice in each experimental group are shown in Fig. 1e.

### TGF-β1 induces WNT10A expression in fibroblast cells

**Fig. 2:**
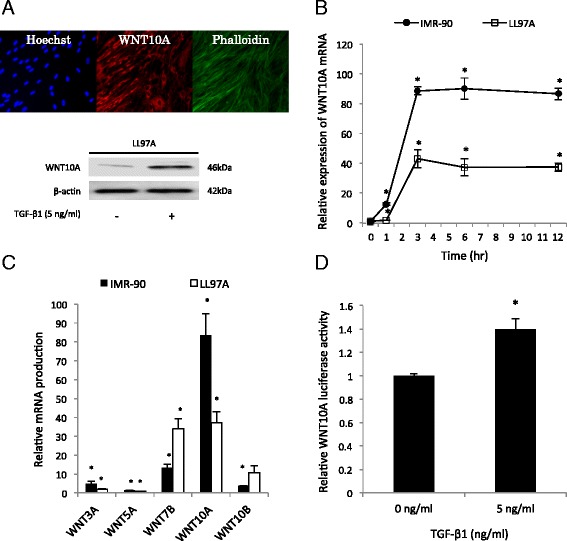
TGF-β1 induces WNT10A expression in fibroblasts. **a** The results of an analysis of Hoechst (blue), WNT10A (red) and Phalloidin (green) staining in LL97A cells as indicated by immunofluorescence. The WNT10A expression after the administration of TGF-β1 (5 ng/ml) in LL97A cells was analyzed using a Western blot analysis. **b** LL97A and IMR-90 cells were treated with TGF-β1 (5 ng/ml), and at the indicated time points, the mRNA level of WNT10A was assessed by real-time qPCR compared to the untreated cells, and the results are expressed as the means ± SEM. ^*^ *p* < 0.05. **c** The WNT family genes (WNT3A, WNT5A, WNT7B, WNT10A, WNT10B and β-catenin) were analyzed by real-time qPCR compared to the untreated cells, and the results are expressed as the means ± SEM. ^*^ *p* < 0.05. **d** The luciferase activity of the WNT10A promoter was measured 24 h after TGF-β1 (5 ng/ml) stimulation, and the results are expressed as the means ± SEM. ^*^ *p* < 0.05

**Fig. 3:**
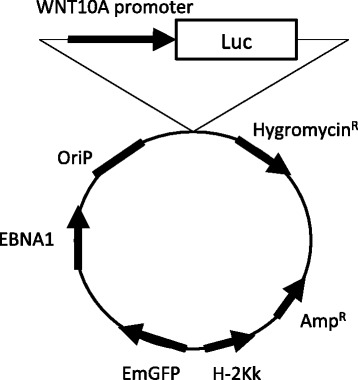
Maps of the plasmid vectors used for the mammalian luciferase reporter assays

To confirm whether TGF-β1 induces WNT10A expression, we used LL97A cells, which were derived from human lung fibroblast cells from IPF patients. As shown in Fig. 2a, the LL97A cells spontaneously expressed WNT10A. In addition, we found that WNT10A expression was activated by TGF-β1 using a Western blot analysis. A real-time qPCR analysis showed that TGF-β1 treatment increased the WNT10A mRNA expression in LL97A cells, as well as in a human diploid fibroblast strain, IMR-90 cells (Fig. 2b). The maximal expression of the mRNA was observed at three hours after treatment. Next, we compared the mRNA expression level of WNT10A and other members of the WNT family, WNT3A, 5A, 7B and 10B, using IMR-90 and LL97A cells. The expression of WNT10A mRNA was induced most strongly by TGF-β1 treatment among these various genes (Fig. 2c). To confirm whether the mRNA expression was increased by effects of transcriptional regulation, we transfected a reporter plasmid containing the WNT10A promoter (Fig. 3) into COS1 cells (fibroblasts from an African green monkey), and a luciferase assay was performed. The WNT10A luciferase activity was increased in the fibroblasts by TGF-β1 administration (Fig. 2d).

### BLM induces WNT10A expression in fibroblasts

**Fig. 4:**
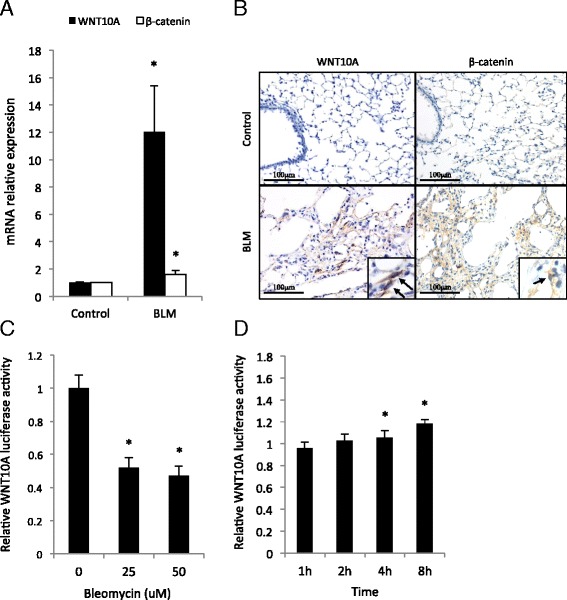
BLM induces WNT10A and β-catenin expressions. **a** The mRNA level of WNT10A and β-catenin in BLM-treated and control mice was assessed by real-time qPCR (*n* = 10 each). *p < 0.05. **b** Lung tissue specimens of BLM-treated and control mice were stained with anti-WNT10A and β-catenin antibodies. The arrow indicates the WNT10A and β-catenin expressions in fibroblasts. **c** COS1 cells stably transfected with WNT10A-luciferase reporter plasmid which can replicate in the cells were treated with the indicated concentration of BLM. After 24 h, a luciferase assay was performed and the results are expressed as the means ± SEM. ^*^ *p* < 0.05. **d** The transfectants described in (**c**) were treated with 50 uM BLM for the indicated times, and then were changed to a new medium. After 48 h, a luciferase assay was performed as a recovery assay, and the results are expressed as the means ± SEM. ^*^ *p* < 0.05

To confirm whether BLM induces WNT10A, we investigated the lungs from BLM-treated mice using real-time qPCR and immunohistochemical analyses. Both the mRNA expression and protein staining of WNT10A in the lungs of BLM-treated mice were increased compared with those in control mice (Fig. 4a and b). To confirm whether this induction was also observed in vitro, COS1 cells transfected with a WNT10A reporter plasmid were used. Unfortunately, the COS1 cells died after sustained treatment with BLM, and the luciferase activity was not increased (Fig. 4c). However, if highly-concentrated (up to 50 uM) BLM was used, short-term treatment induced WNT10A promoter activity (Fig. 4d).

### WNT10A expression induces collagen production

**Fig. 5:**
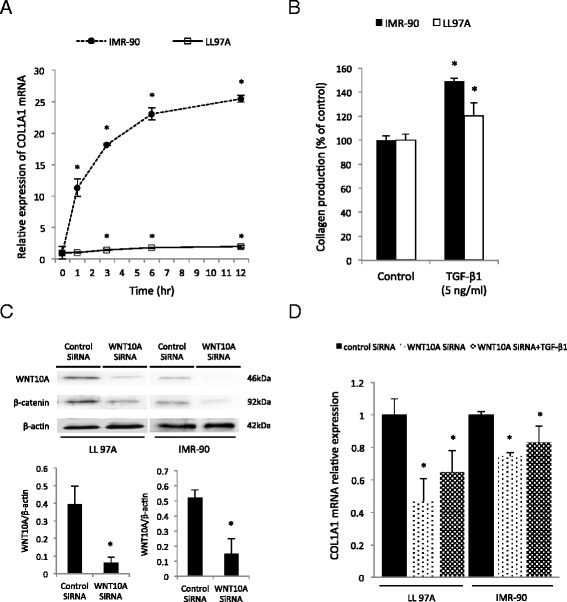
The WNT10A expression regulated collagen production. **a** Cells were treated with TGF-β1 (5 ng/ml), and at the indicated time points, the mRNA level of COL1A1 was assessed by real-time qPCR. ^*^ *p* < 0.05. **b** Collagen production was measured 24 h after TGF-β1 (5 ng/ml) stimulation. ^*^ *p* < 0.05. **c** The efficacy of WNT10A silencing was evaluated by immunoblotting for β-catenin and WNT10A proteins in IMR-90 and LL97A cells. ^*^ *p* < 0.05. **d** LL97A and IMR-90 cells transfected with indicated siRNAs for 72 h were treated with TGF-β1 (5 ng/ml). After 24 h, the mRNA level of COL1A1 was assessed by real-time qPCR. ^*^ *p* < 0.05. All results are expressed as the means ± SEM

To elucidate the mechanisms underlying the role of WNT10A in pulmonary fibrosis, we focused on collagen, one of the components of the extracellular matrix. As shown in Fig. 5a, the COL1A1 mRNA expression levels of IMR-90 and LL97A cells were increased by TGF-β1 treatment in a time-dependent manner. We also investigated the quantity of total collagens in fibroblasts treated with TGF-β1. The total collagen level was significantly increased by treatment with TGF-β1 in both cell line (Fig. 5b). To confirm whether WNT10A was associated with the collagen expression, we employed a RNAi technique (using siRNA). As shown in Fig. 5c, WNT10A expression was strongly decreased by treatment with a siRNA against WNT10A in both fibroblast cell lines. Consistent with a previous report [16], β-catenin expression was reduced with the decrease in WNT10A. The expression of COL1A1 mRNA in both fibroblast cell lines was decreased by treatment with WNT10A siRNA, even if the cells had been activated by TGF-β1 (Fig. 5d).

### Patient characteristics

To investigate the clinical implications of WNT10A overexpression, we obtained lung tissue samples from 30 IPF patients with a histological UIP pattern who underwent a VATS lung biopsy. There were 25 males and five females with a mean age of 70.7 years old. These patients with IPF were divided into two groups (a WNT10A-positive group [*n* = 13] and a WNT10A-negative group [*n* = 17]) as stated previously. No significant differences were observed in clinical variables between the groups (Table 1). Among the 30 patients with IPF, 10 (33.3 %) patients experienced an AE-IPF, including nine patients (90 %) with AE-IPF within a year from VATS. Fifteen patients (50.0 %) died during the observation period, including six (40.0 %) due to acute exacerbation, four (26.7 %) due to respiratory failure, four (26.7 %) due to cancer and one (6.7 %) due to other known causes. The median survival period for patients with IPF was 1,240 days from VATS.

**Table 1:** Comparison between the two groups of IPF patients: positive and negative for WNT10A immunostaining

Characteristic	Positive group (*n* = 13)	Negative group (*n* = 17)	*p*-value
Sex, male, No	11	14	0.782
Age, year, mean	70	71.2	0.711
Brinkman Index, mean	856.9	943.8	0.773
BMI, kg/m^2^, mean	24.9	22.3	0.103
PaO_2_, Torr, mean	80.5	85.2	0.363
SpO_2_, %, mean	97.6	96.9	0.263
FVC, L, mean	2.79	2.88	0.651
% FVC, %, mean	87.3	86.7	0.934
FEV1.0 %, %, mean	73.3	75.3	0.967
% DLCO, %, mean	63.3	65.2	0.662
KL-6, U/ml, mean	1016.6	747.2	0.563
SP-D, ng/ml, mean	168.4	157.7	0.779
LDH, IU/L, mean	235.7	222.5	0.934
MMRC, mean	1.15	1.35	0.509

Data are presented as the n and means

### Survival analysis using the Kaplan-Meier method

**Fig. 6:**
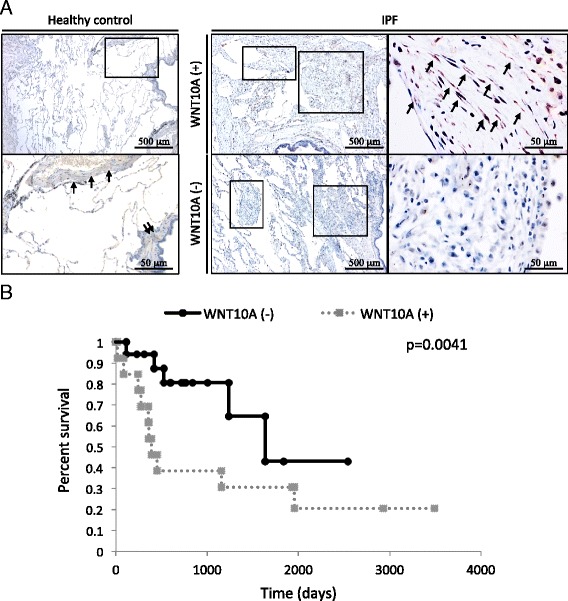
The clinicopathological significance of WNT10A expression in patients with IPF. **a** An immunohistochemical analysis of the WNT10A protein expression in the healthy control and patients with IPF. The left panels show normal human lung tissue treated for the localization of WNT10A using immunohistochemistry reactivity in the smooth muscle of vessels (single arrows) and airways (double arrows). The right upper panels show a WNT10A-positive case, which demonstrated a specific expression of WNT10A in one part of the spindled cells (fibroblasts, indicated by arrows in a high-power view of the right panel) at a site of pulmonary fibroblastic foci (boxes, low-power view of left panel). The right bottom panels indicate a WNT10A-negative case in patients with IPF. **b** The results of the Kaplan-Meier survival analysis of the overall survival in IPF patients who were WNT10A-positive or negative

To confirm whether WNT10A is an effective prognostic factor for patients with IPF, we stained human lung tissue samples with anti-WNT10A antibody. As shown in Fig. 6a, WNT10A protein was observed to be focally expressed in pulmonary fibroblastic foci. A significant difference was noted between the positive and negative groups in terms of the survival time (log-rank, hazard ratio [HR] 5.351, 95%CI 1.703-16.82; *p* = 0.0041) (Fig. 6b). Among the patients with positive WNT10A staining, eight patients (61.5 %) experienced an AE-IPF within a year from the VATS.

### Role of WNT10A as a prognostic factor for acute exacerbation of IPF

**Fig. 7:**
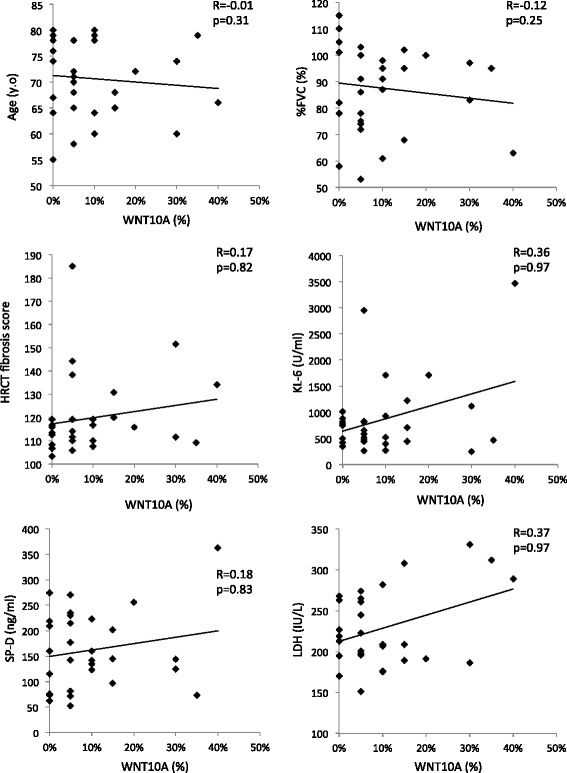
The results of the correlation analysis between WNT10A and the clinical variables in patients with IPF. The Spearman correlation coefficients between these variables were determined as shown on the plots

**Fig. 8:**
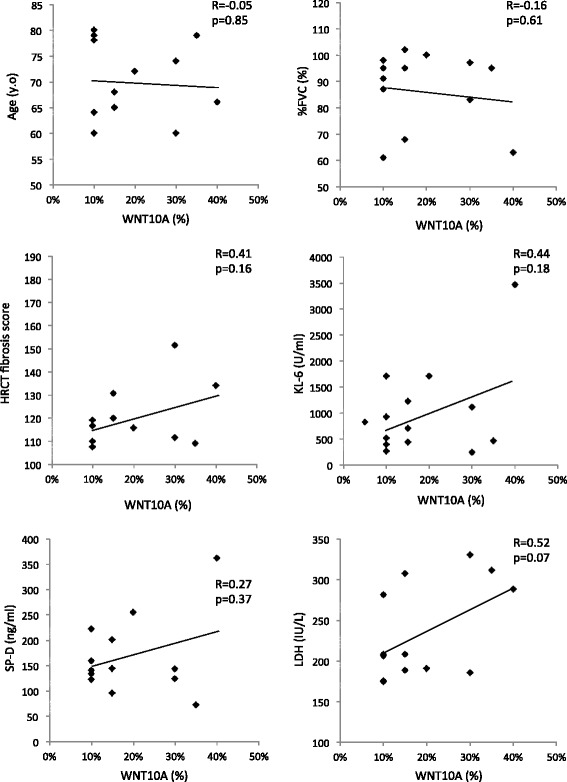
The correlation between WNT10A and the clinical variables in patients with IPF in the WNT10A-positive group. No significant correlations were noted between these clinical variables and positivity of WNT10A based on the Spearman’s correlation coefficients

To investigate the risk factors for an acute exacerbation of IPF, we analyzed the clinical variables (sex, age, %FVC, KL-6, SP-D, LDH, modified Medical Research Council [MMRC] score, HRCT fibrosis score [17] and WNT10A) using a multiple logistic regression analysis. The correlations between the baseline WNT10A expression and clinical variables are shown in Figs. 7 and 8, and were determined using Spearman’s rank correlation coefficient. There was no correlation between the expression of WNT10A and other clinical variables. The results of the multiple logistic regression analysis are shown in Table 2. The overexpression of WNT10A was identified as the only variable significantly predictive of acute exacerbation (Odds ratio [OR] 13.69, 95 % CI 1.728-108.5; *p* = 0.013).

**Table 2:** Logistic regression analysis for the factors predicting AE-IPF

Variables	*p*-value	OR (95 % CI)
WNT10A	0.013	13.69 (1.728 to 108.5)
LDH	0.088	1.022 (0.997 to 1.047)

## Discussion

IPF is a chronic, progressive and fibrosing disease characterized by the proliferation of fibroblasts with deposition of type I collagen within the alveolar wall, which results in scarred non-functional airspaces, hypoxia and death due to respiratory failure [18]. We herein report that TGF-β1-induced WNT10A expression leads to the production of collagen in fibroblasts. By performing in vitro and in vivo experiments, we provided evidence that these mediators are induced at the mRNA level, as well as at the protein level. In addition, WNT10A is a sensitive predictor for the onset of AE-IPF, and its expression is associated with an increased risk of mortality in patients with IPF. To the best of our knowledge, our data provide the first evidence of a link between WNT10A and the development of a fibrosis response in IPF. Therefore, WNT10A may represent a novel pathway contributing to the persisting phenotype of lung fibrosis.

TGF-β is a major inducer of EMT and a key mediator of fibrosis in the lung, and it also activates a large number of intracellular signaling cascades [19]. TGF-β signaling predominantly occurs through the Smad-dependent pathways; however, there is evidence showing that TGF-β induces the activation of non Smad-dependent signaling in EMT [20], particularly regarding the interactions between TGF-β and the β-catenin pathway [21]. In addition, TGF-β signaling can lead to β-catenin signaling through a WNT-dependent pathway. Recent evidence has suggested that WNT signaling, especially the canonical WNT/β-catenin pathway, may be involved in the onset of TGF-β-mediated fibrosis [13].

**Fig. 9:**
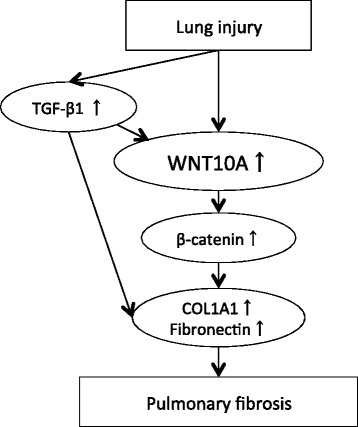
The role of WNT10A in lung fibrosis. A proposal model depicting the role of WNT10A in lung fibrosis is shown. Injury leads to increased WNT10A expression, either directly or via TGF-β1 signaling, in fibroblasts. Aberrant activation of WNT10A is involved in mediating the fibrogenesis in patients with IPF by inducing the accumulation of collagen and fibronectin

WNT10A has multiple functions in regulating cell properties, such as proliferation [22, 23], and we also previously reported that WNT10A overexpression is strongly related to fibrosis in the organs outside the lung [11, 12]. Although prior studies have suggested that several WNT ligands play a central role in epithelial and mesenchymal proliferation and collagen production and enhanced gene expression in patients with IPF [8, 10, 24, 25], no previous studies have so far reported the clinical implications of WNT10A expression in patients with IPF. Several reports have found that the upregulation of TGF-β signaling induces an increase in WNT3A [26], WNT5A [27, 28], and WNT7B [29]. In the present study, overexpression of WNT10A was largely related to an upregulation of collagen production via TGF-β signaling (Fig. 9). As a result, smad binding elements (SBEs), containing the 5′-AGAC-3′ element [30], are important for understanding the relationship between WNT10A and TGF-β. In fact, the 5′-AGAC-3′ SBE was observed at two different spots within 200 bp from the transcriptional start point of the WNT10A gene the present study (data not shown). We believe that these findings indicate a close relationship between WNT10A expression and the upregulation of TGF-β signaling.

β-Catenin is a key component for the alveolar epithelium in lung development [31], where it exists as a component of the adherens junction, and it is bound to E-cadherin, and also is found in the cytoplasm where it induces signaling functions [32]. The role of β-catenin in the lung is less clear, but some studies have demonstrated that a relationship may exist between the expression of β-catenin and pulmonary fibrosis. Kim and colleagues demonstrated that the intratracheal administration of siRNA targeting β-catenin also caused a decrease in lung fibrosis after BLM [33]. In response to TGF-β1, β-catenin is liberated from the E-cadherin adherens junctions and thereafter translocates to the nucleus where it mediates the activation of the transcription factors promoting collagen transcription [34]. In contrast, Tanjore and colleagues demonstrated that β-catenin in the alveolar epithelium protects against BLM-induced fibrosis [35]. In the present study, we focused on the expression of WNT10A in fibroblasts, and showed that WNT10A activated by TGF-β1 is associated with the production of collagen, therefore, further investigation is necessary to clearly elucidate the role of β-catenin.

The extracellular matrix, such as collagen plays a central role in the onset of lung fibrosis [36]. We found WNT10A to be expressed in regions containing fibroblasts and WNT10A is therefore implicated in the production of collagen. In addition, we showed that markedly increased levels of COL1A1, which indicates the presence of collagen synthesis, were observed to decrease thereby reducing the activity of WNT10A in vitro. IPF is characterized by the unrelenting progression of fibrotic tissue formation, with the expansion of fibroblasts within the alveolar walls and the deposition of type I collagen, thus resulting in the obliteration of airspaces and the subsequent impairment of gas exchange that in turn leads to progressive hypoxia [15, 37, 38]. Therefore, our data indicate that inhibiting WNT10A might be an effective approach to target fibrosis in patients with IPF.

The molecular mechanisms underlying AE-IPF are poorly understood and little is known about the sensitive biomarkers for predicting acute exacerbation. Previous studies have reported several genetic factors associated with mortality in IPF, such as erythrocyte complement receptor 1 [39], telomerase reverse transcriptase [40] and the MUC 5B gene [41]. In addition, previous studies also have reported several biomarkers to be significantly higher in AE-IPF than in stable IPF [42–44]. However, none of these markers were found to be associated with the risk of AE-IPF. One previous report suggested that the baseline serum KL-6 level, a complex sialo-carbohydrate glycoprotein present in the human MUC1 mucin, may be a sensitive predictor for the onset of AE-IPF [45]. In our study, the baseline KL-6 level was not found to correlate with AE-IPF; however, patients with a WNT10A overexpression did have a significantly poor prognosis and the WNT10A expression was thus identified to be a sensitive predictor for AE-IPF. The molecular mechanisms underlying AE-IPF remain poorly understood, although Collard and colleagues suggested that stretch-dependent TGF-β activation may promote the development of AE-IPF during VATS [3]. Our findings suggest that WNT10A overexpression via the TGF-β signal leads to an acceleration of fibroproliferation, such as that observed in AE-IPF. In fact, WNT10A overexpression was found to be the only risk factor for AE-IPF according to the findings of a multivariate analysis in the present study.

## Conclusions

Our results show that the WNT10A expression therefore plays an important role in pulmonary fibrosis via TGF-β1 signaling. As a result, inhibiting WNT10A might be a novel approach to treat patients with IPF. In addition, the WNT10A expression may also be a sensitive predictor for the onset of AE-IPF.

## Abbreviations

AE-IPF: acute exacerbation of idiopathic pulmonary fibrosis
BAL: bronchoalveolar lavage
BALF: brochoalveolar lavage fluid
BLM: bleomycin
COL1A1: collagen, type I, alpha 1
EMT: epithelial-mesenchymal transition
GAPDH: glyceraldehyde-3-phosphate dehydrogenase
H&E: hematoxylin and eosin
IPF: idiopathic pulmonary fibrosis
MCP: monocyte chemotactic protein
MEM: minimum essential medium
qPCR: quantitative polymerase chain reaction
ROC: receiver operating characteristic
SBES: smad binding elements
SPSS: statistical package for social sciences
TGF: transforming growth factor
UIP: usual interstitial pneumonia
VATS: video-assisted thoracoscopic surgery
